# Peer play network in preschool classrooms: Features and variations by child characteristics and skills

**DOI:** 10.1371/journal.pone.0327023

**Published:** 2025-08-14

**Authors:** Ji-Young Choi, Ziye Wen, Ashley Boros, Tzu-Jung Lin, Becky Huang, Chia-Hsin Yin

**Affiliations:** 1 Department of Human Sciences, The Ohio State University, Columbus, Ohio, United States of America; 2 Crane Center for Early Childhood Research and Policy, The Ohio State University, Columbus, Ohio, United States of America; 3 Department of Educational Studies, The Ohio State University, Columbus, Ohio, United States of America; 4 Department of Teaching and Learning, The Ohio State University, Columbus, Ohio, United States of America; Free University of Bozen-Bolzano: Libera Universita di Bolzano, ITALY

## Abstract

Peers play a critical role in creating opportunities for learning. However, less is known about how children form peer play networks in preschool and how their level of connection in peer play varies based on individual characteristics and skills. This exploratory study used social network modeling to examine variations in peer play networks across preschool classrooms (*N* = 5), focusing on children’s play centrality and classroom network density. Data from all classroom children, including those with both active and passive parental consent, revealed similar peer play patterns at both mid-year (*N* = 81) and year-end (*N* = 79). Most children had one or more peers with whom they played frequently or always, but a small subset (3%) rarely or never engaged with playmates. Classroom network density fell within a moderate range, suggesting that children tended to select specific peers for play rather than interacting uniformly with all classmates. Analyses of play centrality among children with active consent (*n* = 26) revealed that dual language learners (DLLs), defined as children exposed to a language other than English at home, were less likely than their non-DLL peers to hold central positions in classroom play networks at the end of the preschool year. Additionally, the study identified a homophily effect related to problem behaviors, indicating that children were more likely to play with peers who displayed similar levels of problem behaviors. These findings highlight the importance of recognizing variations in peer play networks to foster inclusive and supportive peer play environments in early childhood education. This study underscores the need for further research into the factors that shape peer play networks in preschool settings, particularly the roles of language, culture, and behavior in influencing children’s interactions.

## Introduction

Preschool-aged children in the United States (U.S.) spend a substantial amount of time in early care and education settings [1], where many experience formal group interactions for the first time. Recognizing play as a vital mechanism for learning during the early years [2], preschool curricula often emphasize a play-based, hands-on approach that provides ample opportunities for peer interactions [3,4]. For example, nearly one-third of the day in an average U.S. preschool is dedicated to free choice play [5], which is a setting found to be associated with more positive peer engagement [6]. Peers become increasingly important at this age as children begin to form and navigate peer relationships through social play and interactions [7]. While solitary play remains common, play involving peer interaction is increasingly observed during the preschool years [8,9]. Empirical evidence has shown that most preschool-aged children establish one or more mutual friendships [10] and engage in more verbal interactions with peers than with adults in the classroom [11].

Peers play a significant role in providing opportunities that support and foster development, making them a crucial component of the classroom environment [12–15]. Positive peer interactions, characterized as children’s social exchanges and subsequent behavioral responses through communication and play, are associated with better child outcomes in areas of social, language, and cognitive development [16–19]. Developmental theories align with these empirical findings, underscoring the important role of peers in early development. Vygotsky’s sociocultural theory [20] highlights how children learn through social interactions with more capable peers, particularly within their zone of proximal development. Building on this, Wood and colleagues [21] introduced the concept of scaffolding, where a more skilled individual provides temporary support to facilitate the learning process. In addition, Bandura’s social learning theory posits that learning can occur through observing behaviors from others [22], where peer modeling and subsequent imitation can facilitate children’s learning. These theories suggest how peer interactions can facilitate learning through scaffolding and modeling behaviors.

In early childhood education (ECE), peer interactions have traditionally been studied at the individual or, at most, dyadic level, often overlooking the complex interconnections among all children in the classroom. Recent studies recognize that dyadic peer interactions and play are interdependent, influenced by broader classroom networks where multiple interactions occur simultaneously [e.g., 23]. This approach acknowledges that peer play functions within a classroom system, where interactions between two children can influence peer interactions with and among other children [24]. Small but emerging empirical evidence from preschool classrooms has further shown that peer social network indicators, such as interconnectedness among peers and social positions in peer interactions, are related to academic achievement [25].

Building on emerging efforts to study child peer interactions within the classroom system, this study examined peer play networks in preschool classrooms. This study aimed to describe these networks and extend recent social network research by exploring how individual child characteristics and skills relate to their peer play networks within preschool classrooms.

### Peer networks based on child characteristics and skills

The ECE literature has long recognized individual differences in the frequency and nature of children’s peer play and interactions [9,26]. These variations have been found to be linked to both child characteristics and skills. For example, studies have shown that older children, girls, and children with an easy temperament tend to engage in more peer interactions [27,28]. Additionally, children with reactive temperaments, problem behaviors, and weaker language skills are often observed to interact less with peers [16,29,30].

Peer social network studies have further demonstrated that individuals’ network centrality, conceptualized as the extent to which individuals are more influential than others within a network [31], can vary based on children’s characteristics and skills, such as disability status [23] and behaviors [32]. For instance, Chow and colleagues’ study [32] found that kindergarteners with higher levels of teacher-reported externalizing behaviors were less central in their classroom’s friendship network, as identified by their peers. Research on language development and disorders further indicates that children with developmental language disorders, those at risk for language impairment, and those with weaker language skills are less likely to occupy central roles in peer social networks [33,34].

Building on prior studies, this study examined how peer social networks differ by child characteristics and skills in preschool classrooms. Of particular interest was dual language learner (DLL) status—defined as children exposed to a language other than English at home. ECE literature has documented that DLLs often experience differential, and at times less supportive, preschool environments [35–37]. For example, a recent study by the LENA team [36] found that in classrooms with English-monolingual teachers, DLLs engaged in 7.5 fewer conversational turns per hour and experienced language isolation at a rate four times higher than their English-monolingual peers.

Although much of the focus on DLLs’ classroom experiences has centered on interactions with teachers, their experiences with peers, particularly in classrooms dominated by English monolinguals, may differ significantly from those of their non-DLL peers. As Tabors [38] noted, learning a new language requires being socially accepted by the speech community; however, to be socially accepted, one must first be able to speak the language. Thus, English language proficiency, often associated with DLL status, could be associated with reduced interactions with peers.

Even when DLLs are proficient in English, they may still face challenges integrating into peer groups in English-dominant classrooms. Research has shown that children, including DLLs, tend to prefer native speakers over children with accents [39,40]. Play is shaped by culture, meaning children from different cultural backgrounds may have differing preferences for activities (e.g., nonsocial vs. sociable activities) and varying cultural norms surrounding social interactions [41]. For example, children from East Asian backgrounds, such as those in Japan and Korea, often participate in group-oriented play that emphasizes social harmony and respect for others, rather than assertive or highly individualistic interactions [42,43]. In Western cultural contexts, on the other hand, American parents tend to view play as a purposeful activity for developing children’s cognitive and social skills that are important for children’s future success [44]. Italian families are reported to value a lively and emotionally expressive style, meaning that even when playing alone, children may display expressive and emotionally rich behaviors influenced by their parents’ cultural values [45]. These differences illustrate that a child’s cultural background significantly influences not only the types of play they engage in but also how they perceive and enact social relationships during play. These also indicate that DLLs who navigate two linguistic and cultural environments may differ from their non-DLL peers, influencing their peer interactions and play dynamics.

Building on the emerging literature on peer social networks in ECE, where understanding of peer play networks based on children’s DLL status remains limited, this study explored how children’s characteristics and skills relate to classroom peer play networks, with a particular emphasis on DLL status.

### Homophily in peer play networks

Studies of peer social networks have further indicated that preschoolers with similar characteristics and skill levels are more likely to interact with one another than those with less similar characteristics and skill levels. Such homophily in peer interaction has been reported based on child sex [46,47], disability status [23,48,49], language skills [50,51], prosocial behaviors [51,52], problem behaviors [53,54], and school competencies [51,55]. For example, Lin et al.’s study^51^ of children in rural preschool classrooms found that stronger dyadic relations developed when children share similar language and literacy skills, suggesting selective peer interactions based on these shared skills. They further found that these interactions became more apparent over the school year, and children demonstrated more skills like peers of their selected interactions than peers with whom they interacted less, adding support for peer homophily based on skills.

Current research offers limited insight into homophily in young children’s expressive language skills, particularly in typical preschool settings. Fine-grained aspects of expressive language, such as linguistic complexity and diverse word use, remain largely underexplored. This study aimed to address this gap by investigating potential homophily in English expressive language skills—specifically variations in word use, vocabulary richness, and syntactic complexity—within children’s play networks. In addition, we examined more commonly studied domains, such as social skills and problem behaviors. These expressive language and social-emotional skills were selected for their malleability and their critical role in supporting children’s learning in school environments. By exploring these areas, the study seeks to offer preliminary insights into how expressive language and social-emotional behaviors uniquely contribute to the formation of peer networks.

### Current study

This exploratory study aimed to characterize peer play networks in preschool classrooms. First, we described two indicators of play networks at mid-year (January–early March) and year-end (April) of the preschool year: (a) children’s peer play centrality and (b) classroom play network density. Peer play centrality indicates the frequency of peer play interactions that a child experiences with peers in the classroom, reflecting their social position (or centrality) with class peers. High centrality indicates that a child is positioned centrally among peers and serves as the hub of the social network, while low centrality suggests that a child is more isolated or less connected within the classroom. Classroom play network density represents the overall level of connectedness within the classroom, with higher density indicating stronger and more frequent connections among the children.

Then, we examined child characteristics and skills that explain variations in the child-level peer play networks (i.e., peer play centrality) at the end of the preschool year. This examination considered attributes at both the individual child level and the dyad level. At the individual level, we focused on DLL status along with child age, sex, developmental delays, and sibling status, as well as social skills, problem behaviors, and English expressive language skills. At the dyad level, we explored the similarity or homophily effect of English expressive language and social-emotional skills (i.e., social skills and problem behaviors) between dyads in the classroom.

In summary, the study addressed the following research questions:

1. How can peer play networks (play centrality and classroom play network density) in preschool classrooms be characterized at mid-year and at the end of the school year?
2. What factors predict peer play centrality by the end of the school year?
  2-1. Do child characteristics and skills, particularly DLL status, predict peer play centrality?
  2-2. Do children who share similar levels of skills in English expressive language, social skills, and problem behaviors engage in play more frequently?

Expanding on the traditional approach in which interactions and relationships are studied at the individual or dyadic level, this study adopts a classroom-wide perspective that accounts for the complex, interconnected nature of peer play, which recognizes that children’s interactions are influenced within broader social networks where multiple interactions occur simultaneously.

## Methods

### Participants

This study was conducted in an urban area of the Midwest, U.S. Between 8/11/2023 and 2/22/2024, nine preschool teachers and 82 children were approached for recruitment. The final analytic sample included 26 preschool children (57.7% female; 51.81 months old [SD = 6.78] in mid-year) from five preschool classrooms within three centers. The consent rates were 55.56% for teachers and 31.7% for children (26 out of 82 children; 12% to 100% consent rate per classroom). Between January and April, two children dropped out of their classrooms, and no new attendees joined during this period. Both of the children who left had been part of the passive consented participants. Approximately 50% of parents identified themselves as White, 34.6% as Black, 11.5% as Asian or Pacific Islander, and 3.8% as multiracial.

Out of the 26 participating children, four children from four classrooms were reported by parents to be exposed to a non-English language at home, with varying degrees of English exposure. The primary spoken language of the children ranged from ‘predominantly their home language’ to ‘exclusively English.’ According to parent reports, three of the four DLLs demonstrated excellent skills in both speaking and listening in English, while one child was reported to have less than adequate proficiency in these areas. In terms of home language skills, three DLLs were rated as excellent in speaking and listening, while one child was rated as poor in both domains. Among the four DLL children, two were identified as Asian, one as Black, and one as multiracial.

All teachers reported that English was the primary language of instruction in their classrooms. Teachers across five classrooms reported a total of 10 children who were exposed to a non-English language at home (ranging from 0 to 5 DLLs per classroom), representing eight different home languages: Hindi, Japanese, Korean, Mandarin, Marathi, Spanish, Taiwanese, and Telugu.

### Recruitment and data collection procedures

Using convenience sampling, we identified and contacted 13 centers; three center directors granted permission for recruitment of teachers and families within their centers. With the directors’ permission, team members visited consenting centers to recruit preschool teachers. All lead teachers of preschool classrooms were given packets containing study details, a consent form, and an initial teacher survey to complete. Of the nine teachers contacted across the three centers, five consented to participate. Shortly after submitting written consent, teachers received a $50 e-gift card for study compensation. An online teacher survey option was available to all teachers through a QR code that led to Qualtrics. Two lead teachers completed the survey online, with the remaining three utilizing paper copies. After collecting all surveys, the paper copies were manually added to the Qualtrics by a team member.

The project manager worked with participating teachers to send home parent packets via backpack mail that contained study details, a consent form, and the parent survey to invite them to consent to their child’s participation in the study. All recruitment materials and parent surveys were made available in both English and Spanish. Shortly after submitting written consent, parents received a $25 e-gift card via email. Participating parents completed an initial parent survey asking questions about their family and child in this study. An online survey option was available through an included QR code that led to Qualtrics. Approximately 19% of the participating parents completed the online version, with the remaining 81% completing a paper copy. A team member manually added all paper copy surveys into our Qualtrics database.

With the help of the directors, the project manager sent paper copies of opt-out forms to all non-participating parents. These forms allowed non-participating parents to request that their child’s information be excluded from teacher-reported peer play interaction surveys. No parents returned the opt-out form, giving passive consent for the five participating teachers to report on all children’s play interactions in their classrooms.

Data were collected from five classrooms across two waves during the 2023–2024 preschool year: mid-year (January–early March) and year-end (April). Two classrooms were recruited in the fall (October–November) and had additional data collected in the fall, while three classrooms were recruited in the winter and only had data from two waves, excluding fall data. We used data from both the mid-year and year-end collections to maximize the data available for this study. Throughout all data collection waves, teachers completed child reports on paper, focusing on peer play interactions and social-emotional behaviors. The research team coordinated classroom visits with the assistance of directors and teachers to assess children’s English expressive language skills at mid-year and year-end.

### Ethics statement

This research was approved by the authors’ Institutional Review Board (2023B0148). Teachers provided written consent to participate. Written parental consent was obtained for all children who actively participated in the study. Verbal assent was also obtained from each child prior to the direct child assessments. Passive consent was used for non-participating children, allowing their data to be excluded from teacher-reported peer play interaction surveys if parents opted out. No parents returned the opt-out form, providing passive consent for the five participating teachers to report on all children’s play interactions in the five classrooms.

### Measures

#### Child characteristics.

Parents completed a survey asking basic child and family characteristics, including the child’s age, race/ethnicity, identified hearing problems and developmental delays, sibling status and respective age/s, and primary language children hear and speak at home at the time of recruitment. Parents from homes where language/s other than English was spoken reported on their child’s proficiency in both English and their home language.

#### Peer play network.

Teachers reported the frequency of play between pairs of children across all children in the classroom using the Teacher-Rated Peer Interaction (TRPI) scale [23]. When taking the scale, teachers were asked to consider children’s play behaviors (e.g., pretend play or sharing toys) from the past three months. Teachers reported each pair of children’s frequency of play interactions from 0 to 4: 0 (never play), 1 (rarely play), 2 (sometimes play), 3 (often play), and 4 (always play). Reported play interactions were presumed symmetrical or undirected so that teachers only had to rate each pair of interactions once. These ratings were used to calculate child play centrality and classroom play network density within and across participating classrooms.

#### Social skills and problem behaviors.

Teachers rated focal children’s social skills and problem behaviors using two subscales of Social Skills Improvement System Rating Scales (SSIS-RS; [56]): Social Skills and Problem Behaviors. SSIS-RS has been widely utilized in early childhood research to examine social-emotional behaviors in children between the ages of 3 and 18 [57]. Teachers were directed to think about the child’s behavior over the past 2 months and rate the frequency of each focal child’s social skills (e.g., “Follows your directions”) and overall problem behaviors (e.g., “Is aggressive towards people or objects”) in their classrooms, using a 4-point scale (1 = never, 2 = seldom, 3 = often, 4 = almost always). The test-retest reliability estimates were.82 and .92, respectively [56].

#### English expressive language skills.

Trained team members assessed English expressive language skills one-on-one using a story-retelling task. The story-retelling task was selected because it prompts children in this age group to produce significantly more word tokens, word types, and utterances [58]. A popular wordless picture book, *Frog Where are You* [59] was used for the task at year-end. Children began by listening to audio narrations of the stories, which were sourced from the SALT software website [60], while simultaneously viewing the accompanying picture books. Children were then asked to retell the stories using picture books as visual aids [e.g., 46,61,62]. All recordings of retellings were transcribed verbatim by trained research team members. All recordings were initially transcribed by one researcher and verified by another. The transcripts were segmented into standard communication units [C-units; 63] and coded following the Human Analysis of Transcripts conventions of the Child Language Data Exchange System [CHILDES; 64]. The Computerized Language Analysis (CLAN) program [64] was then utilized to derive three expressive language measures: (a) *types*, a measure of different words used, (b) *lexical (vocabulary) diversity*, a measure of the overall range of vocabulary used considering the frequency of types relative to the total words used, and (c) *Mean Length of Utterance in morphemes (MLU-morphemes)*, a measure of syntactic complexity derived from dividing the total number of morphemes by the total number of utterances in each retell.

### Analytical approach

Data analysis was conducted in two phases. In phase one, we transformed the play network data collected from the TRPI for all children in the classroom into a symmetrical matrix, a data structure more conducive to network analysis. This matrix was imported into R using the igraph package [65] for the construction and visualization of individual classroom networks and the calculation of a centrality score for each child. Two variables of the peer play network were derived: (a) Standardized play centrality and (b) classroom play network density. Standardized play centrality, a measure of each child’s social position in the classroom network [66], indicates the frequency at which a child plays with others in the classroom. To account for differences in classroom sizes, individual play centrality was standardized by dividing the raw score by the maximum possible number of social ties in the classroom, calculated as classroom size (n) minus 1. This maximum considers both the number of peers and the strength of ties, with strength values also ranging from 0 (never play) to 4 (always play). Higher centrality scores indicate that a child plays with more peers more frequently, suggesting a stronger social influence in the classroom’s peer play network. In contrast, lower scores indicate fewer and less frequent peer interactions. To estimate the overall classroom-level interconnectedness of peer play within the classroom, we used weighted classroom play network density. Classroom play network density was calculated as the sum of tie strengths in the classroom divided by the maximum possible tie strengths (assuming all ties are at their highest value of 4 “always play”). Classroom play density was rescaled to range from 0 (no peer play) to 1 (all children *always* playing together). To address research question 1, to characterize peer play networks in preschool classrooms at mid-year and year-end, preliminary data analysis was conducted on the standardized play centrality and classroom play network density variables based on data collected at both time points.

Next, the standardized play centrality and classroom play network density variables were merged into the master dataset, which included the characteristics and skills of the focal children whose parents consented to participate in this study (*N* = 26). These included age, sex, developmental delays, sibling status, DLL status, as well as social skills, problem behaviors, and English expressive language skills. For research question 2−1, to examine which child characteristics and skills predict peer play centrality, we first conducted correlation analyses among standardized play centrality, social skills, problem behaviors, and expressive language skills (MLU-morphemes, indicating syntactic complexity) at the end of the school year. Second, linear regression models with Generalized Estimated Equations [GEE; 67] were used to analyze the clustered data (i.e., children nested within classrooms), with child characteristics including DLL status, English expressive language skills (MLU-morphemes), social skills, and problem behaviors as the predictors.

For research question 2−2, dyadic correlation analysis was performed to explore the homophily effect. Each child dyad was categorized based on their TRPI ratings. Dyads with a rating below 3 (0 = never play, 1 = rarely play, 2 = sometimes play) were grouped into the “low play frequency” category, while dyads with a rating of 3 or higher (3 = often play, 4 = always play) were grouped into the “high play frequency” category. A correlation analysis was then conducted separately for each group to examine whether the target outcome variables for children within each dyad were correlated. The outcome variables included English expressive language skills (Types, Vocabulary Diversity, and MLU-morphemes, separately), social skills, and problem behaviors.

## Results

### Descriptive statistics

Descriptive statistics of the study sample are shown in Table 1.

**Table 1:** Descriptive statistics of child characteristics and skills.

Variable	*N* (%)	Mean (SD)	Median	Min-Max (Range)
*Child characteristics*				
Sex				
Male	11 (43.3)			
Female	15 (57.7)			
DLL Status				
DLL	4 (15.4)			
Monolingual	22 (84.6)			
Sibling				
Has sibling(s)	8 (30.8)			
Does not have a sibling	18 (69.2)			
Developmental Delay				
Yes	3 (11.5)			
No	23 (88.5)			
Age (mid-year)	26	51.81 (6.78)	50	39-63 (24)
*Child skills* (year-end)				
Social Skills	26	2.87 (.30)	2.93	2.15-3.39 (1.24)
Problem Behaviors	26	1.83 (.43)	1.98	1.20-2.73 (1.53)
MLU Morphemes	26	6.40 (2.04)	6.34	8.47 (1.35-9.82)

*Note. N* = sample size; SD = Standard Deviation; Min–Max = Minimum and Maximum scores.

DLL = dual language learner.

Descriptive statistics for the peer play network are presented in Table 2. There was a total of 704 child play dyads in mid-year and 678 child dyads in the year-end of preschool. Among these, 18.6% of dyads “never” engaged in play interactions at mid-year, decreasing to 9.59% at year-end. Over 90% of children (*n* = 73 in mid-year and *n* = 74 year-end) played with at least one peer “often” or “always,” and about 3% (**n* *= 2) “rarely” or “never” played with any peers in the classroom in both time points.

**Table 2:** Descriptive statistics of peer play frequency.

Variable	*N* (%)	Mean (SD)	Median	Min-Max (Range)
*Peer play frequency (Child)*				
Mid-school year	81	1.81 (.62)	1.80	.4-2.84 (2.44)
End of the school year	79	1.80 (.44)	1.90	.71-2.68 (1.98)
*Peer play frequency (Dyad)*				
Mid-school year				
Never	131 (18.6)			
Rarely	176 (25)			
Sometimes	217 (30.82)			
Often	117 (16.62)			
Always	63 (8.95)			
*Total dyad*	704			
End of the school year				
Never	65 (9.59)			
Rarely	249 (36.73)			
Sometimes	195 (28.76)			
Often	135 (19.91)			
Always	34 (5.01)			
*Total dyad*	678			

*Note. N* = sample size; SD = Standard Deviation; Min–Max = Minimum and Maximum scores.

### Peer play network

#### Peer play centrality.

The play centrality, reflecting the frequency of peer play interactions, was below “2 = sometimes” on average in the middle of the school year (*M* = 1.81, SD = 0.62) and at the end of the school year (*M* = 1.80, SD = 0.44). This suggests that, on average, children occasionally played with other peers in the classroom. The correlation of play centrality between the two time points was relatively high, at 0.72 (*p* < 0.10). This suggests that the frequency of peer play interactions remained relatively stable, though not entirely unchanged, over the approximately 2–3-month period. Since classroom sizes varied, individual play centrality was standardized by dividing the raw score by the maximum possible number of social ties in the classroom, calculated as the classroom size (n) minus one. This standardized value was used in the subsequent analysis.

#### Classroom play network density.

Classroom play network density ranged from 0.33 to 0.57 across classrooms at both mid-year and year-end (see Table 3). In other words, 33% to 57% of dyadic play interactions were formed out of the maximum possible interactions that can be formed in the classroom. No consistent pattern was found in changes in classroom play network density between mid-year and year-end, as the network density decreased in three classrooms and increased in two classrooms.

**Table 3:** Classroom play network density in mid and end of the preschool year *(N*_*classroom*_* *= 5).

Classroom	Play network density		Class size		# of changes in children between January and April	
	Mid-year	Year-end	Mid-year	Year-end	Dropout	New enrollee
1	.568	.486	12	11	1	0
2	.363	.452	16	15	1	0
3	.545	.518	8	8	0	0
4	.332	.367	25	25	0	0
5	.562	.505	20	20	0	0

*Note.* Play network density measures the degree of interconnectedness of peer play interactions within each classroom. It ranges from 0 (no peer play) to 1 (all children always interacted with each other), with higher scores indicating more connected peer interactions. Classroom network density values were weighted by play interaction frequency, ranging from 0 (never) to 4 (always), to reflect both the number and intensity of connections. Then, those values were rescaled to range from 0 (no peer play) to 1 (all children *always* playing together).

### Peer play network in relation to child characteristics and skills

Bivariate correlations among key variables are presented in Table 4. Standardized play centrality was significantly correlated with children’s DLL status, indicating that DLLs had lower centrality (*r* = –.46, *p* < .05). No other significant correlations were found between standardized play centrality and the other variables examined in this study. Social skills were negatively correlated with problem behaviors (*r* = –.43, *p* < .05) and were higher among children with siblings (*r* = .42, *p* < .05). Girls in our sample were more likely to have siblings (*r* = .44, *p* < .05) and less likely to have developmental delays (*r* = –.42, *p* < .05) compared to boys.

**Table 4:** Bivariate correlation among standardized play centrality, social skills, problem behaviors, and MLU morphemes at the end of the preschool year.

Variable	1	2	3	4	5	6	7	8
1.Sex (1 = female)								
2.Age	−.14							
3.DLL status (1 = yes)	−.07	.21						
4.Sibling status (1 = yes)	.44*	−.20	−.18					
5.Developmental delay	−.42*	.32	−.15	−.02				
6.Standardized play centrality	.13	−.19	−.46*	−.05	−.31			
7.Social skills	.34	−.13	−.11	.42*	−.20	−.04		
8.Problem behaviors	−.15	−.02	−.01	.15	.11	.04	−.43*	
9.MLU-morphemes	.27	.12	−.17	.25	−.08	.21	−.20	.34

*Note.* DLL = dual language learner; MLU = Mean length of utterance (syntactic complexity).

* *p* < .05.

#### Child characteristics and skills as predictors of peer play centrality.

**Fig 1:**
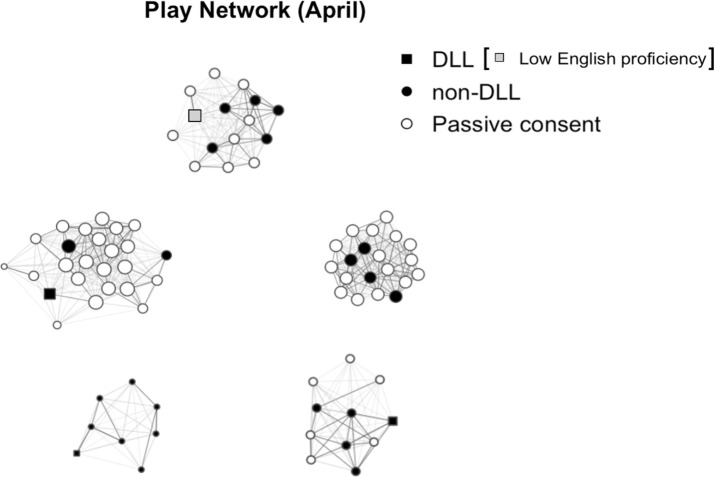
Visual representation of the peer play network at the end of the semester (April). The color and shape of the node indicate dual language learner (DLL) status (Square = DLL; Gery square = DLL with low English proficiency; Circle = non-DLLs), as well as active vs. passive consent status (Black or Gery = participants; Empty circle = children with passive parental consent). The size of the node shows the play interaction, the larger the node, the more play interactions the child had with peers in the classroom; the darkness of the ties between nodes reflects the frequency of play interactions between children based on the teacher rating- the darker the tie, the more frequent the child’s play with each other.

We examined how child characteristics and skills are associated with their peer play centrality within a dyad at the end of the school year (research question 2−1). Results showed that children’s DLL status was the only predictor of play centrality near the end of the school year, with DLLs showing a lower centrality in peer play than their English monolingual peers (*β* = −.58, *p* < .001; Table 5). As shown in Fig 1, DLL children were less likely to be situated at the center of classroom play networks compared to their English monolingual peers. All other child characteristics, including sex, age, sibling status, and developmental delay, as well as child skills, including social skills, problem behaviors, and MLU in morphemes (expressive English language), were not significantly associated with peer play centrality (Table 5).

**Table 5:** Generalized estimated equations: Child characteristics and skills Predicting standardized play centrality at the end of the school year (*N*_*child*_ = 26).

Variable	*β* (SE)
*Model 1*	
Sex (1 = female)	−.01 (.14)
Age	.00 (.01)
DLL status (1 = yes)	−.58 (.13)***
Sibling (1 = yes)	−.13 (.21)
Developmental Delay	−.50 (.31)
Social Skills in April	−.10 (.43)
Problem behaviors in April	.03 (.16)
MLU Morphemes	.02 (.05)
*Intercept*	1.103 (1.62)

*Note.* DLL = Dual language learners; MLU = Mean length of utterance (syntactic complexity).

* *p* < .05, ** *p* < .01, *** *p* < .001.

#### Homophily effect.

Children’s play frequency showed significant homophily based on problem behaviors (research questions 2−2; see Table 6). Specifically, significant correlations were found for both the high play frequency group (*r* = .42, *p* < .05) and the low play frequency group (*r* = .38, *p* < .05). This suggests that child play partners are more likely to exhibit similar problem behaviors. The homophily effect was not found in three indicators of English expressive language skills (i.e., types, vocabulary diversity, and MLU-morphemes) and social skills.

**Table 6:** Correlation between play interaction frequency (high vs. low) and target outcome variables at the end of the year in April.

Variable	Low play frequency (*n* = 39)	High play frequency (*n *= 23)
*Language Proficiency*		
Types	−.10	−.20
Vocabulary Diversity	−.18	.25
MLU-morphemes	−.30	.12
*Social-Emotional Behaviors*		
Social Skills	−.09	−.06
Problem Behaviors	.38*	.42*

*Note.* There is a total of 62 child dyads among consented children in April. Child dyads with a rating below 3 (0 = never play, 1 = rarely play 2 = sometimes play) are grouped into the “low play frequency” category, while dyads with a rating of 3 or higher (3 = often play 4 = always play) are grouped into the “high play frequency” category. MLU = Mean length of utterance.

*p < .05.

## Discussion

This exploratory study aimed to characterize the peer play network in preschool classrooms, focusing on peer play centrality and classroom play network density, at mid-year and year-end. We also examined child characteristics and skills associated with children’s peer play centrality at the end of the school year and whether children played more frequently with peers who shared similar English expressive language skills and social-emotional behaviors.

### Peer play network

On average, children in our study played with peers “sometimes” at both mid-year and year-end. The majority of children had one or more peers they played with frequently, although a small number of children did not have any playmates in the classroom. Children’s play centrality in this study was similar to that reported in a study of preschoolers that used the same play interaction scale [23]. This previous study reported a classroom network density of 1.96 on a scale of 0–4, which is comparable to our study’s density range of 0.33–0.57 on a scale of 0 (indicating no play) to 1 (indicating play always occurs among all children). The classroom network density observed in this study was moderate (*M* = .47, SD = .06), indicating that children tended to interact with select playmates rather than engaging equally with all peers. This pattern of selective interaction is consistent with previous research showing that preschoolers often form preferential play relationships rather than fully inclusive peer networks [24,25]. The patterns of play centrality, along with the moderate level of network density, both reflect children’s selective choices of playmates. Notably, these patterns remained stable between the mid-year and end-of-year assessments of the preschool year, spanning an approximately 2- to 3-month period. Our findings, which demonstrate variations in peer play interactions among children within classrooms, suggest potential opportunities to promote more socially integrated peer play in preschool settings. They further raise the question of why such variation in peer play patterns occurs—a question we seek to explore in the following research question.

### Peer play network in relation to child characteristics and skills

Perhaps the most intriguing finding of this study is that children’s DLL status was a predictor of their play centrality at the end of the school year. Among various child characteristics and skills examined, such as sex, developmental delay, social skills, problem behaviors, and expressive English skills (MLU-morphemes), DLL status emerged as the only significant predictor. Specifically, DLL children were less likely to be central in the peer play network, meaning they were less connected in their peer play interactions compared to their English monolingual peers. It is important to note that the parents of three out of four DLL children in this study rated their child’s English speaking and listening skills as “excellent.” We also controlled for expressive English skills in the model. Therefore, their lower centrality in the peer play network could not be solely explained by their English proficiency.

While limited, existing research suggests that DLLs tend to have less stimulating and qualitatively different classroom experiences compared to their non-DLL peers in typical U.S. preschool classrooms, where English is the dominant language, and DLLs often represent a minority group [35–37]. This study contributes to the limited body of research on classroom environments for DLLs, suggesting that DLLs may experience less connected peer play interactions in these settings. It is possible that DLLs use less sophisticated language patterns or display differences in vocabulary, accent, or language structures during peer interactions. Other factors associated with children’s DLL status, such as race/ethnicity and culturally influenced play interests and norms for engaging in social interactions during play, may also play a role in shaping their peer interactions [39,40,68,69]. While the underlying reasons for this are unclear, this study provides initial evidence highlighting the need for teachers’ attention toward DLLs—children exposed to languages other than English at home, regardless of their English proficiency—to ensure equitable learning opportunities in preschool classrooms. Replication of this study with a larger sample size is necessary to validate these findings further.

In this study, we did not find a homophily effect on language or social skills reported in other studies of preschoolers [e.g., 51]. However, we found homophily effects in overall problem behavior, indicating that children tended to play more with peers who exhibited similar levels of problem behaviors. Considering peer effects on problem behaviors [12], it is possible that problem behaviors are reinforced and spread among peers. Children may be drawn to play with peers who display similar levels of problem behaviors. Alternatively, children with higher levels of problem behaviors may have fewer available playmates to choose from and may gravitate toward peers who exhibit similar behaviors, as other children may be reluctant to interact with them. Future studies with an in-depth analysis of specific types of problem behaviors are necessary to understand the underlying causes of homophily in these behaviors within preschool settings.

### Limitations and future directions

Study limitations should be acknowledged to contextualize the results and guide future research. First, this study should be considered exploratory due to the use of convenience sampling, low participation rates, and a small sample size. While convenience sampling is frequently used in the field for its accessibility and cost- and time-efficiency, it limits the generalizability of our findings and underscores the need for future replication studies. In addition, future replication studies of social network analyses with larger child samples, both within and across classrooms, are needed to address limitations related to small sample size and low participation rate in our sample. Second, while this study focused on child-level characteristics and skills as predictors of children’s peer play networks, we acknowledge that peer interactions can also be influenced by classroom-level factors, such as peer composition and the classroom climate for inclusion fostered by teachers. Future studies with larger classroom sample sizes could contribute to the existing literature by exploring how classroom characteristics affect the formation of peer play networks. Third, studies with larger samples of DLLs representing a range of English proficiency, race/ethnic identity, and cultural backgrounds are warranted to identify whether and under what circumstances the current findings can be replicated. Such studies could further clarify potential reasons for differences in peer play interactions based on DLL status.

## Conclusion

Taking a classroom-wide perspective that acknowledges the complex and interconnected nature of peer play, where children’s interactions are shaped within broader social networks involving multiple simultaneous connections, this study examined peer play networks in preschool classrooms. Several key findings emerged from this study, highlighting variations in peer play networks among children in preschool settings. First, we found evidence that children tended to interact with select playmates rather than engaging equally with all peers. Second, English monolingual children were more likely to occupy central positions in classroom play networks, whereas DLLs, children exposed to languages other than English at home, were less central in these networks. Third, a homophily effect was observed for problem behaviors, suggesting that children were more likely to engage in play with peers who exhibited similar levels of problem behaviors.

Although the underlying reasons for these patterns remain unclear, the critical role of peer relationships in early development underscores the need for greater attention to fostering positive peer play interactions. Specifically, this study offers early evidence emphasizing the importance of supporting DLLs in their peer interactions to ensure equitable learning opportunities. Additionally, the findings highlight the need to consider individual child skills, such as problem behaviors, when examining peer dynamics. Overall, these results have important implications for fostering ECE environments that support children with diverse linguistic backgrounds and behavioral skills. These findings also highlight the need for further investigation into the factors that influence peer play networks, particularly how variations in language, culture, and behavior shape children’s social interactions.
